# A Novel Splice Site Mutation in *HPS1* Gene is Associated with Hermansky-Pudlak Syndrome-1 (HPS1) in an Iranian Family

**Published:** 2016-07-03

**Authors:** Soudeh Ghafouri-Fard, Feyzollah Hashemi-Gorji, Vahid Reza Yassaee, Nasrin Alipour, Mohammad Miryounesi

**Affiliations:** 1 *Department of Medical Genetics, Faculty of Medicine, Shahid Beheshti University of Medical Sciences, Tehran, Iran.*; 2 *Genomic Research Center, Shahid Beheshti University of Medical Sciences, Tehran, Iran.*

Sir, 

Hermansky-Pudlak syndrome (HPS) is a rare autosomal recessive disorder which is characterized by oculocutaneous albinism, bleeding, and lysosomal ceroid storage resulted from deficiencies in multiple cytoplasmic organelles including melanosomes, platelet-dense granules, and lysosomes (1). A wide variation has been detected in the phenotypes of patients suffering from this disorder. The amount of pigmentation of the skin, hair, iris and fundus is markedly different among patients with HPS from an almost total absence of pigment to a nearly normal amount. Pigmentation degree possibly increases with age (2). Although the bleeding tendency is mild in these patients, certain evaluations are necessary before any surgery to prevent life-threatening complications (2). Ceroid deposition in these patients would result in pulmonary fibrosis and granulomatous colitis (3) with the former being the main cause of death in HPS after 1 year of age (4). No generalized defect has been detected in peripheral blood lymphocyte or neutrophil function in a group of studied HPS patients (5). However an association has been observed between HPS and lupus or frequent bacterial infections which is attributed to a defect within the monocyte-macrophage system, possibly secondary to ceroid accumulation and decreased activity of natural killer cells, respectively (6).

Several genetic loci have been shown to be associated with different types of HPS. Among them is the *HPS1* gene associated with HPS1 (OMIM #203300) which encodes a transmembrane protein that is believed to be a component of multiple cytoplasmic organelles and is critical for their normal development and function (1). Accordingly, it is a part of a protein complex that controls the intracellular localization of lysosomes and late endosomes and participates in the biogenesis of lysosome-related organelles complex as well as melanosome biogenesis (7).

**Fig 1:**
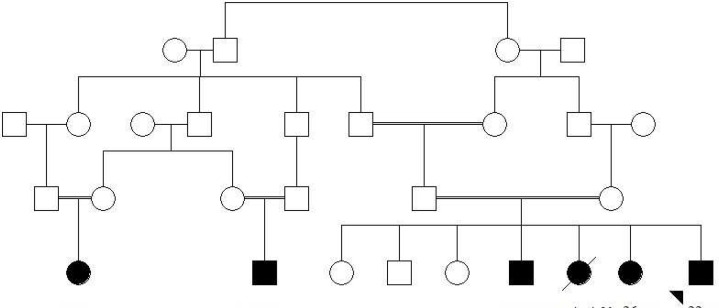
The pedigree of family

**Fig 2:**
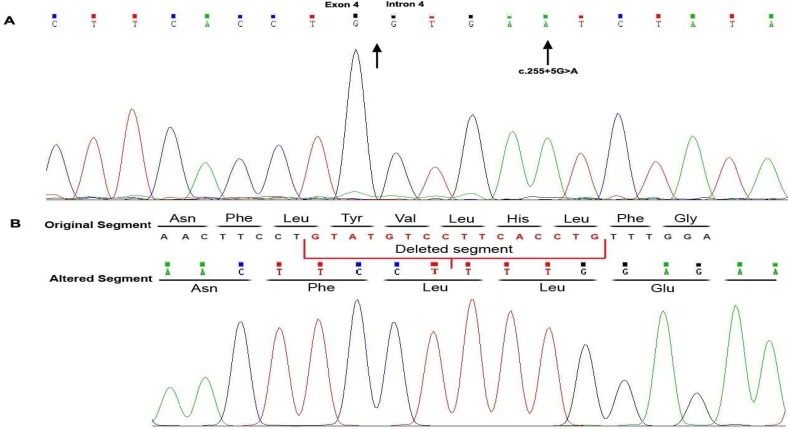
Genomic and cDNA sequences of affected HPS proband. A: nucleotide change at DNA level in homozygous state in the proband. B: transcript sequencing shows a 17 bp deletion and frameshift in patient compared with the original sequence

Here we report a 22-year-old patient presented with generalized skin hypopigmentation, brown hair, reduced visual acuity, photophobia, and nystagmus. He reported a history of easily bruising with minor trauma, without any history of internal organ bleeding. His parents were consanguineous (Figure 1). No symptom or sign of pulmonary disorder was reported or found in his physical examination. However, one of his sisters had a similar phenotype plus lupus like manifestations and died at the age of 26 due to pulmonary fibrosis. No gastrointestinal complication was reported. All affected members of the family had a history of recurrent infections as well as bleeding tendency. None of them reported symptoms of pulmonary involvement except for the demised patient. The pigmentation degree was not significantly different between the proband and his affected sibs. However, two other patients in the pedigree had more severe hypopigmentation. Genomic DNA was extracted from peripheral blood leukocytes of the patient after informed consent using the standard salting out method. Sequence analysis was performed using NimbleGen chip capturing of 15 Albinism related genes including *GPR143*, *MITF*, *OCA2*, *SLC45A2*, *TYR* and *HPS1* followed by next generation sequencing (BGI-Clinical Laboratories, Shenzhen, China). A homozygous mutation was detected in *HPS1* gene (c.255+5G>A) which has not been reported in generalist polymorphism databases (ExaC or exome variant server (EVS)). The results were confirmed by Sanger sequencing in patient. To evaluate the significance of this novel splice site mutation, the full-length *HPS1*-cDNA was synthesized from RNAs extracted from lymphocytes and then amplified by PCR. A fragment of amplified cDNA in the region of exons 4-5 was sequenced using the ABI Prism3130 Genetic Analyzer (Applied Biosystems, Foster City, CA, USA). This novel splice site mutation has been shown to result in a 17 bp deletion and a frameshift afterwards (p.Y81Lfs*38) (Figure 2). Other affected family members did not participate in the study.

Here we reported a novel splice site mutation in *HPS1* gene in an Iranian family suffering from HPS1. The defined mutation has been shown to cause a deletion in the transcribed RNA and subsequent frameshift and truncated protein. Of note, the observed phenotypes were different in affected family members from a severe pulmonary involvement in a single patient which started in her twenties to a relatively normal pulmonary function in others with some of them being beyond such age. In addition, pigmentation degree was different between affected relatives. Such variable phenotypes could be partly attributed to the age of affected individuals but also gene-gene and gene-environment interactions and should be assessed in future studies. Other studies have also reported phenotypic variability in the HPS1 symptoms including colitis (8), pulmonary fibrosis (9) as well as pigmentation degree even between patients having similar mutation (9).

The first homozygous frameshifts in *HPS1* have been found in Puerto Rican, Swiss, Irish, and Japanese HPS patients in 1996. The different clinical phenotypes associated with different *HPS1* frameshifts implied that differentially truncated HPS1 polypeptides may have fairly different significances in subcellular function (10). Previous reports demonstrated a frameshift at codon 322 to be the most common *HPS1 *mutation in Europeans and suggested the presence of a founder effect in Puerto Rican patients. Although other mutations have been reported, a frameshift hotspot has been detected at codons 321-322 (1). However, the detected mutation in the present study is localized in a different location from those reported previously which is in accordance with proceeding reports implying that mutation load for the disease-causing genes may be specific to the Iranian population and cannot be inferred from other populations (11). Although we have demonstrated the consequence of detected mutation at transcript level and predicted its effect at protein level, further researches are needed to explore its importance in subcellular localization of the truncated protein.
